# Unwinding focal segmental glomerulosclerosis

**DOI:** 10.12688/f1000research.10510.1

**Published:** 2017-04-12

**Authors:** Vasil Peev, Eunsil Hahm, Jochen Reiser

**Affiliations:** 1 Department of Internal Medicine, Rush University Medical Center, Chicago, IL, USA

**Keywords:** focal segmental glomerulosclerosis, FSGS, nephrotic syndrome, suPAR

## Abstract

Focal segmental glomerulosclerosis (FSGS) represents the most common primary glomerular disease responsible for the development of end-stage renal disease (ESRD) in the United States (US). The disease progresses from podocyte injury to chronic kidney disease (CKD), ultimately leading to total nephron degeneration. Extensive basic science research has been conducted to unwind the mechanisms of FSGS and, with those insights, understand major contributors of CKD in general. As a result, several putative molecules and pathways have been studied, all implicated in the disease; some serve, in addition, as early biomarkers. The ongoing research is currently focusing on understanding how these molecules and pathways can interplay and be utilized as potential diagnostic and therapeutic targets. Among these molecules, the soluble urokinase plasminogen activating receptor (suPAR) has been studied in detail, both clinically and from a basic science perspective. By now, it has emerged as the earliest and most robust marker of future CKD. Other circulating factors harming podocytes include anti-CD40 auto-antibody and possibly cardiotrophin-like cytokine factor-1. Understanding these factors will aid our efforts to ultimately cure FSGS and possibly treat a larger portion of CKD patients much more effectively.

Focal segmental glomerulosclerosis (FSGS) is seen as a histologic pattern of injury rather than a disease by itself. It represents a condition frequently associated with nephrotic syndrome (NS) in adults and children, particularly in the United States (US). Because of the focal and segmental appearance, it is commonly missed on renal biopsy. Nevertheless, this condition is encountered in 35% of patients with NS who have undergone a renal biopsy, of which 50% represent African Americans (AA) ^1^; thus, it is considered the most common primary glomerular disease in adults leading to end-stage renal disease (ESRD) in the US ^2^. FSGS is noted at a 4-fold higher rate in AA compared with Caucasian and Asian patients and 1.5- to 2-fold higher in males compared to females ^2^. Interestingly, some European countries have reported this condition to be a less common cause of NS ^3^ than appreciated in the US, favoring a certain genetic predisposition of AA patients.

FSGS has been classified in many different ways and mainly driven by its histopathological description. One more broadly accepted classification describes FSGS as primary (or idiopathic) or secondary (adoptive). While primary FSGS accounts for about 40% of idiopathic NS and is frequently associated with a rapid decline of renal function, the secondary form of FSGS more frequently follows an indolent course. Secondary FSGS is characterized by rather non-nephrotic range proteinuria, eventually progressing to a nephrotic range as glomerular failure ensues, mainly as a result of progressive interstitial fibrosis. The etiology of secondary FSGS has been established and includes gene mutations, viruses, toxins, and structural and functional adaptation (e.g. hypertrophy, hyperfiltration, and loss of renal mass), while that of primary FSGS remained elusive until more recently. Intensive research has been conducted in this field, leading to the identification of several promising molecules and pathways that could help unveil the complicated mechanisms of idiopathic FSGS, thereby aiding a cure for this condition.

The podocyte, a key cell involved in the maintenance of a normal filtration barrier ^4^, anchored in the glomerular basement membrane (GBM) by discrete foot processes, appears to be the central cell of injury in most forms of FSGS. The podocytes demonstrate an inability to directly replicate, but novel insights suggest that some replacement is possible ^5^. Once exhausted, podocyte density decreases and nephron loss occurs, leading to focal areas of denudation at the GBM interface ^6^. In addition, molecules like transforming growth factor beta (TGFβ) may accelerate podocyte damage by changing stimulation of the expression of the enzyme cytosolic cathepsin L ^7^. Cytosolic cathepsin L in podocytes has been shown to have the ability of cleaving the large GTPase dynamin ^8^, synaptopodin ^9^, and CD2AP ^7^ in various animal models, some mimicking FSGS. Cleavage of CD2AP results in the release of dendrin as a cathepsin L transcription factor, further enhancing the vicious cycle ^7^. In diabetic nephropathy, cathepsin L-mediated activation of pro-heparanase into activated heparanase is associated with albuminuria and preceded the loss of synaptopodin in a streptozotocin-induced diabetes mouse model. This implies that cathepsin L signaling activation may be an early/upstream event in the development of diabetic nephropathy ^10^. In line with the damaging action of cathepsin L are data showing that allosteric activation of the large GTPase dynamin protects from cathepsin L-mediated cleavage of dynamin and restores renal function in models of diabetic kidney disease and experimental FSGS by extending the survival of mice with genetic deletion of CD2AP ^8^.

An intriguing fact about FSGS lies in its high recurrence rate after kidney transplantation (up to 40%). Because of this phenomenon, it was concluded that a circulating factor(s) in the serum of the transplant recipient could be the culprit of the recurrence and of the native disease. This concept was introduced by Savin *et al*. in a pivotal paper describing the exposure of rat glomeruli to sera from patients with rapidly recurrent FSGS after renal transplantation, which led to increased glomerular permeability for albumin ^11^. The putative permeability factor(s) are bound to protein A and have a molecular size of between 30 and 50 kDa. This study was followed by a clinical case report by Gallon *et al*. demonstrating proof of concept of the involvement of the circulating factor(s) in the pathogenesis of FSGS ^12^. The authors described a case of rapidly recurrent FSGS in a kidney transplant recipient after a living donation and reversal of the patho-histologic findings of FSGS and proteinuria in the same allograft once this was removed from the first recipient and re-transplanted in a different recipient without FSGS as native disease.

To date, several circulating factors have been proposed as possible culprits of FSGS, including the one most studied: soluble urokinase plasminogen activating receptor (suPAR). The uPAR gene was cloned in 1989 by Blasi *et al.* ^13^, and the crystal structure of full-length uPAR was solved in 2005 by Llinas *et al.* ^14^. suPAR represents a soluble (circulating) form of an otherwise membrane-bound three-domain receptor for urokinase that mediates extracellular proteolysis ^15^. The uPAR signaling protein has been shown to be expressed on a variety of cells, including active leukocytes, endothelial cells, podocytes, and, most recently, immature myeloid cells ^16, 17^. suPAR has been linked to the activation of the immune system and cancer and first emerged as a direct pathogenic molecule in FSGS ^18^. A recent study exploring the origin of circulating suPAR, which is elevated in proteinuric kidney diseases such as FSGS, found the main source of elevated, pathological levels of suPAR to be extra-renal and pinpoint bone marrow (BM)-derived immature myeloid cells ^16^. The authors found that Gr-1 ^lo^ immature myeloid cells are markedly increased in the BM of proteinuric animals with high suPAR, and these cells are able to efficiently transmit disease when transferred to healthy mice. Consistently, a humanized xenograft mouse model of FSGS resulted in suPAR-associated proteinuria accompanied by an expansion of mouse Gr-1 ^lo^ immature myeloid cells in BM. These results support the notion that suPAR is a circulating factor of FSGS. Moreover, a novel suPAR transgenic mouse model expressing full-length suPAR allows for the analysis of the kidney-damaging effects of long-term suPAR exposure ^16^ in contrast to short-timed models ^19, 20^, as had been originally suggested ^18^.

While the role of suPAR for FSGS in mouse models has been studied in some models ^16, 18– 22^, the role of different suPAR glycovariants as well as their role in human primary FSGS is currently under further research. The suPAR effect in podocytes has been shown to activate αvβ3 integrin, which plays an important role in the maintenance of the controlled adhesion to the GBM as well as the dynamic regulation of mature foot processes ^17^. In this mouse study, sera from patients with recurrent FSGS, but not from those with non-recurrent FSGS or normal controls, activated β3 integrin activity *in vitro*, promoting cell motility and activation of the small GTPases cdc42 and Rac1, while inhibition of suPAR reduced β3 integrin activity and reduced podocyte motility *in vitro* in addition to proteinuria reduction in these animals ^17^. On the other hand, the expression of non-integrin-binding mutant forms of suPAR did not induce glomerular disease. Additionally, studies suggest that suPAR is a regulator of uPAR/uPA actions through competitive inhibition of uPAR, and several studies conclude that the cleaved receptor is a chemotactic agent promoting the immune response ^23^. Studies have demonstrated that plasmapheresis, commonly used to treat recurrent FSGS after transplantation, can lead to the induction of clinical remission. Decreases of both serum suPAR levels and β3 integrin activity in cultured human podocytes served as a bioassay for better renal survival in a subset of patients with recurrent FSGS ^18, 24^. Higher concentrations of suPAR before transplantation were found to confer an increased risk for recurrence of FSGS after transplantation, even in patients with advanced CKD. In patients with preserved renal function, suPAR levels were found to be elevated in two-thirds of subjects with primary FSGS ^18^. Levels of suPAR greater than 3,000 pg/ml seem to correlate strongest with the likelihood of having idiopathic (and possibly recurrent) FSGS in both adult and pediatric patients, as demonstrated in a study where suPAR levels were measured using the commercially available assay in pediatric and adult patients with preserved eGFR ^25^.

Similar findings were observed in a large Chinese cohort, where patients with the most severe histologic injury had the most prominent suPAR elevation and those who responded to therapy and attained remission had lower levels ^26^. Of note, suPAR’s negative correlation with eGFR is lost in patients with eGFR above 90 ml/minute ^27^. The correlation of suPAR with GFR, especially in patients with advanced CKD and ESRD, caused some initial confusion in the field. As a result, it was not clear if serum suPAR elevations are a cause or consequence of renal disease. The initial studies by Wei *et al.* used sera from patients with preserved renal function but some of the follow up studies didn’t. As a result, it was concluded by some that suPAR is not a reliable clinical marker for FSGS across the whole GFR range but is only in patients with GFR above 40 ml/minute ^28^. These issues needed further study. A large two-cohort prospective observational study demonstrated that elevated serum suPAR levels precede the decline of renal function (independently of baseline GFR) and associate with faster progression of CKD ^27^. This finding of suPAR predicting future CKD development (in an independent fashion from baseline GFR) was validated by Guthoff *et al.* ^29^ as well as Schultz *et al.* ^30^. The former study sought to define the positive predictive value of suPAR for albuminuria development in type 2 diabetic patients. The study analyzed the relationship of baseline suPAR and incident microalbuminuria in a prospective long-term cohort of subjects at increased risk for type 2 diabetes (TULIP, n = 258). A higher baseline suPAR was associated with an increased risk of new-onset microalbuminuria in subjects at risk for type 2 diabetes (hazard ratio 5.3 [95% CI 1.1–25.2, *P* = 0.03] for the highest versus the lowest suPAR quartile) and predicted the onset of microalbuminuria as the first clinical sign of renal involvement in this cohort by several years before routine laboratory testing would become abnormal. Here, the increased suPAR levels were upstream of CKD development and before the GFR decline. Furthermore, a significant interaction between time and baseline suPAR suggested that the effect increased over time ^29^. This finding sheds light on the possibility of suPAR being more than a sole biomarker of disease but rather a pathogenic factor for CKD development (FSGS included) whose detrimental effect(s) on the kidney becomes more and more apparent with increasing period of exposure to elevated suPAR levels, something in line with suPAR transgenic animal experiments ^16^. Further corroboration of suPAR’s predictive role for CKD came from a study by Schulz *et al.* showing that suPAR predicted CKD incidence and kidney-related hospitalizations in healthy subjects up to 19 years earlier ^30^.

Additional circulating factors that have been implicated in the development of FSGS include anti-CD40 auto-antibody and cardiotrophin-like cytokine factor-1 (CLCF1). In brief, the CD40 molecule has been shown to be involved in immunity and inflammation. It is expressed in various tissues, including on the surface of B lymphocytes, macrophages, monocytes, dendritic cells, and endothelial and epithelial cells. The binding of the CD40 ligand to the respective receptor leads to increased expression of chemokines, metalloproteases, uPA, and suPAR ^31, 32^. Recently, a study by Delville *et al.* implicated the utility of anti-CD40 antibody measurement for the prediction of recurrent FSGS after renal transplantation ^22^. In this study, the authors analyzed the ability of a panel of ten antibodies against glomerular antigens to reliably predict recurrent FSGS after transplantation. The authors concluded that a panel of seven antibodies (CD40, PTPRO, CGB5, FAS, P2RY11, SNRPB2, and APOL2) can predict the recurrence of post-transplant FSGS with 92% accuracy. Pre-transplant elevation of anti-CD40 antibody was found to have the best correlation and predicted recurrent FSGS with 78% accuracy. Patients with recurrent FSGS were also found to have altered immunogenicity of the extracellular CD40 domain via epitope mapping. Interestingly, the anti-CD40 antibody purified from recurrent FSGS sera did not detect recombinant human CD40 but disrupted the actin cytoskeleton of the human podocytes *in vitro*. This points towards a likely post-translational modification of the native CD40 molecule *in vivo* that is necessary for detection with anti-CD40 antibody. Mechanistically, the authors showed significant involvement of the suPAR-αvβ3 integrin pathway in anti-CD40 antibody-associated proteinuria. The injection of anti-CD40 antibody from recurrent FSGS patients into wild-type mice was not sufficient to cause significant albuminuria in the absence of co-administration of full-length suPAR. An antibody against suPAR or a small molecule targeting the activation of αvβ3 integrin blocked the effect of anti-CD40 antibody/suPAR on human podocytes ^22^. Therefore, the development of molecules that block the activation of αvβ3 integrin may lead to the impairment of anti-CD40 antibody/suPAR signaling and have potential therapeutic implications. Currently, anti-CD40-blocking antibodies (ASKP1240 or lucatumumab) are commercially available and are being tested in clinical trials ^33^.

Cardiotrophin-like cytokine factor-1 (CLCF1) is a member of the IL-6 family with an estimated molecular weight of 22 kDa. CLCF1 is believed to be secreted and is found in the circulation as a heterodimeric composite cytokine with either cytokine receptor-like factor-1 (CRLF1) or soluble receptor alpha for ciliary neurotrophic factor (sCNTF Rα) ^32^. Absent an original report, a study by Savin *et al.* was described as part of a review article that reported isolation of CLCF1 from the active plasma fraction of patients with recurrent FSGS. The authors found the concentration of CLCF1 to be 100-fold higher in the affected individuals compared to concentrations of the factor measured in healthy controls ^34^. The same group demonstrated that incubation of murine podocytes with CLCF1 led to disruption of the actin cytoskeleton in a time- and concentration-dependent manner, resulting in a motile phenotype of the podocytes ^32, 35^. The authors further investigated the effect of either monomeric CLCF1 or the CLCF1-CRLF1 heterodimer on the permeability of rat glomeruli. Additionally, they studied the interaction of these two forms of the circulating factor with key elements of the JAK/STAT pathway. In their key experiments, they compared the effect of CLCF1 with that of sera from FSGS patients on glomerular albumin permeability *in vitro* using anti-CLCF1 antibody or inhibitors of JAK2 and STAT3. Their results demonstrated that while monomeric CLCF1 or FSGS serum increased the albumin permeability of rat glomeruli, the heterodimer CLCF1-CRLF1 attenuated this effect *in vitro*. Furthermore, they found that commercially available JAK2 or STAT3 inhibitors effectively blocked the increasing glomerular permeability effect of CLCF1 or serum from FSGS for albumin *in vitro*. They concluded that future research should focus on studying the role of CLCF1 and related molecules in the etiology of recurrent FSGS as well as consider the application of JAK2 and STAT3 inhibitors in the treatment of FSGS ^36^.

In summary, there seems to be growing potential in the development of drugs targeting one or more circulating permeability factors that now have more established roles in the pathogenesis of FSGS. SuPAR is a circulating biomarker for future renal disease and progression of CKD. SuPAR may be the causative agent if it appears in relatively higher serum levels of full-length suPAR (measured by ELISA), if it is present together with its isoforms, or if it is partnered with other injury molecules (such as anti-CD40 auto-antibody). The latter scenario probably favors the pathogenesis of FSGS over more general forms of CKD. These factors need to be studied further, as they harbor great promise to unwind FSGS and CKD and their targeting may provide novel and safe treatment options ^37^.
